# Study on Shielding and Radiation Resistance of Basalt Fiber to Gamma Ray

**DOI:** 10.3390/ma15072522

**Published:** 2022-03-30

**Authors:** Baoming Ding, Lei Zhang, Jiaqi Liu

**Affiliations:** 1 School of Earth Sciences and Resources, China University of Geosciences, Beijing 100083, China; cugbding@163.com; 2 Institute of Geology and Geophysics, Chinese Academy of Sciences, Beijing 100029, China; manxuenuc@126.com

**Keywords:** basalt fiber, gamma ray, mass loss, shielding and radiation resistance, XCOM

## Abstract

In this study, four basalt rocks were selected to produce continuous fibers, the chemical composition of basalt rocks and the corresponding fibers were compared using the XRF, the results reveal that the content of the chemical component present in the basalt fibers is consistent with basalt rocks. The mass attenuation coefficient of different fibers was analyzed using the XCOM program, the results indicate that when the incident electron energy is 0.01~0.1 MeV, fiber mass attenuation coefficient is found to be positively correlated with the content of Wt (Fe_2_O_3_ + MnO + TiO_2_ + CaO + K_2_O). The structure and properties of the fibers irradiated by different absorption doses of gamma rays were studied using the SEM, EDX and FTIR, the results indicate that irradiation produces no effect on the basalt fiber structure, surface morphology, and contents of the surface elements, the mass loss rate of the fiber was much less than 1%, fiber tensile strength and elastic modulus increased 4.7–7.5% and 3.9–9.1%, respectively, but the elongation at break of fiber decreased 4.18–10.97%. Two selected basalt fiber cloths of thickness 0.12 and 0.28 mm were irradiated with gamma rays of energies of 100 and 120 keV to examine the shielding property of basalt fibers against the gamma rays, when the energy was 100 keV, the shielding ratios of the fiber cloths were 18.9% and 22.5%, respectively, but when the energy was 120 keV, the shielding ratios of the fiber cloths decreased significantly and were at 8.7% and 10.4%, respectively. When the irradiated electron energy is 100 keV, the shielding ratio for basalt fiber cloths measuring 0.12 and 0.28 mm can reach up to 38.9% and 46.3% of that of the 0.5-mm lead plate, respectively.

## 1. Introduction 

Continuous basalt fiber (CBF) is derived from natural basalt ore melted at high temperature and drawn at high velocity through a platinum–rhodium alloy plate, which is another high-performance fiber after glass fiber, carbon fiber, and aramid fiber, and possesses several remarkable properties, such as high and low temperature resistance, flame retardance, fire resistance, corrosion resistance, radiation protection, and excellent mechanical properties [1,2,3]. CBF has a wide range of applications in many fields, such as fire protection, environmental protection, construction, transportation, aviation, and aerospace [4,5,6,7]. CBF is an inorganic silicate material composed of multiple oxides and contains elements of larger atomic numbers, such as iron, titanium, and manganese [8,9], so it exhibits a radiation-proof property. The constant advancement of nuclear science and technology has led to increasingly higher requirements on the performance of radiation-resistant materials. The requirements have shifted from traditional materials, such as iron, tungsten, lead, and concrete to composite shielding materials with several desirable properties, such as good structural properties, high temperature resistance, good mechanical properties, decent shielding properties, lightweight, non-toxic, and malleability [10,11,12]. CBF has been widely used in the field of antiradiation material in recent years because of its excellent comprehensive properties [13,14,15,16,17]. Meanwhile, a great deal of research has been done about CBF reinforce the radiation resistance of composite material, for example, Isfahani et al. [18] studied the properties of clay minerals utilized for disposing of radioactive waste after basalt is added to them in various proportions, and experimental results have revealed that the addition of CBF can significantly enhance the radiation shielding property of the corresponding clay minerals; Ran et al. [19] investigated the effects of gamma ray irradiation on the properties of basalt fiber-reinforced epoxy resin matrix composites and realized that the composites supplemented with basalt fiber possessed considerable advantages in mechanical property retention rate over the mechanical property of the reported polymer. Yassien et al. [20] studied the characteristics of basalt fiber after subjecting it to gamma ray irradiation with total absorbed doses of 3, 10, 25, and 40 kGy. The results shown that gamma ray irradiation produced no effect on the physical structure and chemical properties of the basalt fiber. Nonetheless, effects of chemical composition of CBF on radiation properties and the changes occurring in the mechanical property of the fibers before and after the irradiation have not been studied in this paper.

Having said all of the above, the existing researches mainly focus on basalt fiber reinforced composites, although there are some research on basalt fiber itself, there are no in-depth studies. Different oxides possess different mass attenuation coefficients [21]. Studies have shown that the increased content of oxides, such as TiO_2_, Fe_2_O_3,_ and K_2_O, can greatly strengthen the gamma ray shielding property of the glass or alloy materials [22,23,24]. CBF is mainly composed of SiO_2,_ TiO_2_, Al_2_O_3_, FeO\Fe_2_O_3_, MgO, CaO, K_2_O, Na_2_O, and P_2_O_5_. Different basalt ores produce CBF with different oxide content, as a result that radiation resistance of CBF may be different. In this paper, we selected four kinds of basalt with different chemical compositions, and prepared continuous fibers with filament diameters of approximately 8.0–9.6 µm in the laboratory using the basalt fiber full electric fusion experimental wire drawing furnace. We used the XCOM program to analyze the mass attenuation coefficients of different CBF, meanwhile, the methods to improve the radiation resistance of basalt fiber were discussed based on the influence of different oxides on the fiber production process. The surface morphology, structure, element distribution, and mechanical property variations of basalt fiber before and after the irradiation were investigated via radiation treatment of different fibers with using ^60^Co as the radiation source. Furthermore, Basalt fiber cloths of thickness 0.12 and 0.28 mm were selected for gamma ray shielding ratio performance tests, and compared with a lead plate with a thickness of 0.5 mm.

## 2. Experiments

### 2.1. Materials

#### 2.1.1. Basalt Fiber Preparation

**Figure 1:**
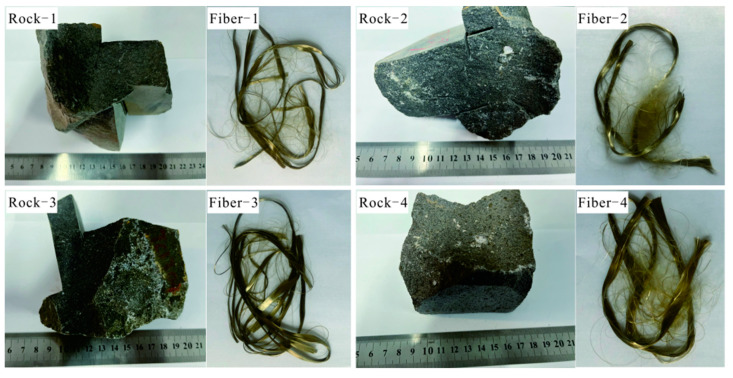
Basalt and corresponding fibers.

Four basalt rocks were selected as raw materials, and the electric-melting basalt fiber single-hole wire drawing furnace was used for fiber preparation to obtain fiber without sizing (Figure 1), four basalt rocks were selected from northeastern China. The fiber manufacturing process includes crushing samples, melting samples, and wire drawing; specifically, firstly, the raw basalt materials were cleaned and dried and then crushed into samples with a diameter of about 5 mm. Subsequently, the crushed samples were put in a clean platinum–rhodium alloy single-hole wire drawing crucible, and then transferred into a fully electric melting single-hole wire drawing furnace. Afterwards, the temperature control program was initiated, increasing the furnace temperature to 1400 °C (±1 °C) in accordance with the set temperature gradient, while the furnace temperature was maintained constant until the sample in the crucible completely melted. Finally, the wire drawing experiment was conducted, in which the furnace temperature was regulated with a temperature gradient of ±5 °C to find a suitable wire drawing temperature range for the corresponding sample until a continuous basalt fiber was extracted. All of the fiber samples for this study were prepared at 1350 °C. The densities of basalt fiber samples are 2.73, 2.76, 2.75, and 2.77 g/cm^3^, respectively.

#### 2.1.2. Basalt Fiber Cloth

**Figure 2:**
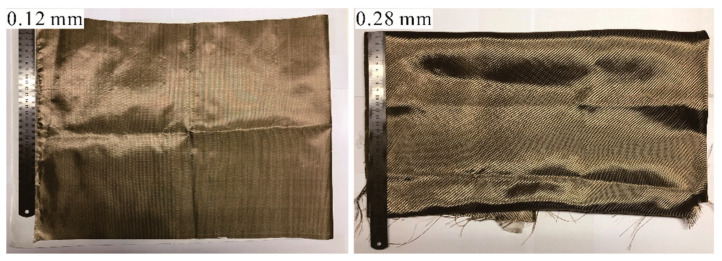
Basalt fiber cloths.

The shielding property of the basalt fiber against gamma irradiation was evaluated using basalt fiber plain cloths of 0.12 and 0.28 mm thickness (the fiber used to make the basalt fiber cloth was formed by direct melt drawing of basalt ore raw materials without the addition of special components). The fiber cloth was provided by Huierjie Corporation of Hubei, China (Figure 2). The density of the basalt fiber cloth is 2.75 g/cm^3^.

#### 2.1.3. Sample Treatment

Four prepared fiber samples were subjected to irradiation with ^60^Co radiation sources at total absorbed doses of 50, 100, and 150 kGy, respectively. The gamma ray energy used was 1.173 MeV, the radiation dose rate was 6 Gy/h, the temperature was room temperature, and the total absorbed dose error was less than 1%.

The basalt fiber shielding property was tested using gamma rays with an electron energy of 100 and 120 keV at room temperature. 

### 2.2. Test and Analyses Methods 

The chemical compositions of the raw basalt materials and the corresponding elements used to prepare the basalt fiber were tested with an X-ray fluorescence spectrometer (XRF, XRF-1500, Shimadzu Corporation, Tokyo, Japan), the accuracy of analysis is better than 5%.

The gamma radio resistance of the basalt fibers with different chemical compositions was analyzed using the XCOM program [25].

The mechanical properties of the basalt fibers were tested with single fiber tensile tester (XQ-1A, Shanghai, China). The test method is in accordance with the standard ASTM C1557-14. The sample gauge length and loading velocity was 25.0 mm and 2.0 mm/min, respectively. At least 30 sets of valid data were measured for each sample, which were then averaged and standard deviations recorded.

The weight of the basalt fiber was weighed using an electronic balance with a recorded accuracy of 0.0001 (CP114, OHAUS, Shanghai, China). Each fiber sample was tested 10 times, which were then averaged and standard deviations recorded. The fiber mass loss rate before and after the irradiation treatment was calculated using Equation (1):

(1) $$M_{loss}=100\%\times (M_{1}-M_{2})/M_{1}$$

where *M_loss_* is mass loss rate, *M*_1_ is fiber mass before radiation, and *M*_2_ is fiber mass after radiation.

Scanning Electron Microscope (SEM, JEOL JSM-7500 F, Tokyo, Japan) was used to observe the changes in fiber surface morphology before and after the gamma irradiation treatment. Energy Dispersive X-ray Micro-analysis (EDX, Oxford Instruments, Oxford, UK) was adopted to test the content of the elements on the fiber surface. The point scanning method was used to test the elemental content at a certain position on the fiber surface. Infrared spectrum characteristics of the fibers were obtained using Fourier Transform Infrared Spectroscopy (FTIR, PerkinElmer, Fremont, CA, USA), and test spectral records ranged between 4000 to 500 cm^−1^, in order to reduce noise interference, each sample was scanned 50 times, the resolution is better than 0.16 cm^−1^. The ionization chamber detector (ALOKA ICS-323C, Tokyo, Japan) was used to detect the energy variations in 100 and 120 keV gamma rays after passing through the basalt fiber cloth. The shielding ratio of the basalt fiber was calculated using the Equation (2):

(2) $$R=100\%\times (I_{0}-I_{t})/I_{0}$$

where *R* is shielding ratio, *I*_0_ is Readings of the tester when the gamma ray does not pass through the fiber cloth, and *I_t_* is Readings of the tester after gamma ray passes through the fiber cloth.

## 3. Results and Discussions

### 3.1. Chemical Composition of Basalt Fiber

The experimental results (Table 1) revealed that for the component contents of the raw basalt materials and the corresponding fibers, except for LOI, the main oxide components are essentially consistent. The reduction in LOI is caused by the volatile constituent significantly reduce content after the basalt is re-melted at high temperatures during the fiber preparation. The main component of the fiber is SiO_2_, followed by Al_2_O_3_, Fe_2_O_3_, CaO, and MgO, where these account for about over 90% of the total content.

**Figure 3:**
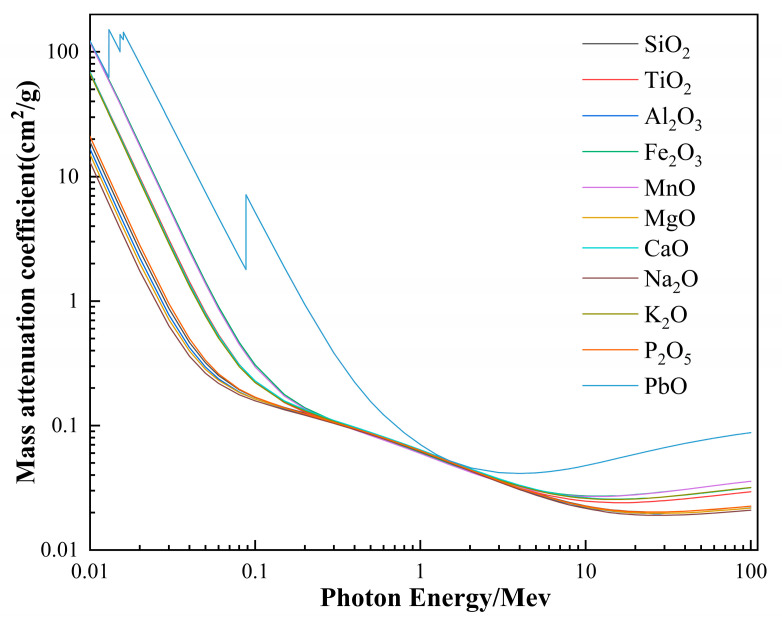
Basalt fiber oxide and PbO mass attenuation coefficient.

**Figure 4:**
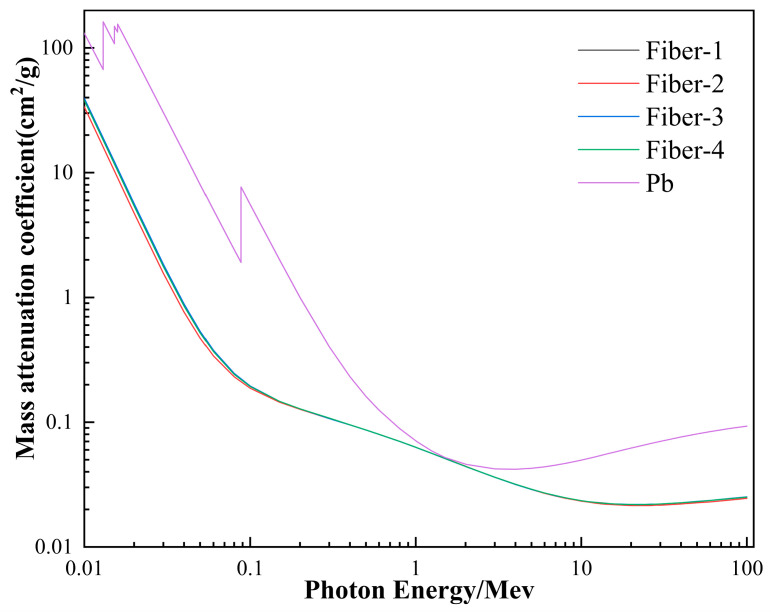
Obtained fiber mass attenuation coefficient according to different oxide content (Pb as a comparison).

**Figure 5:**
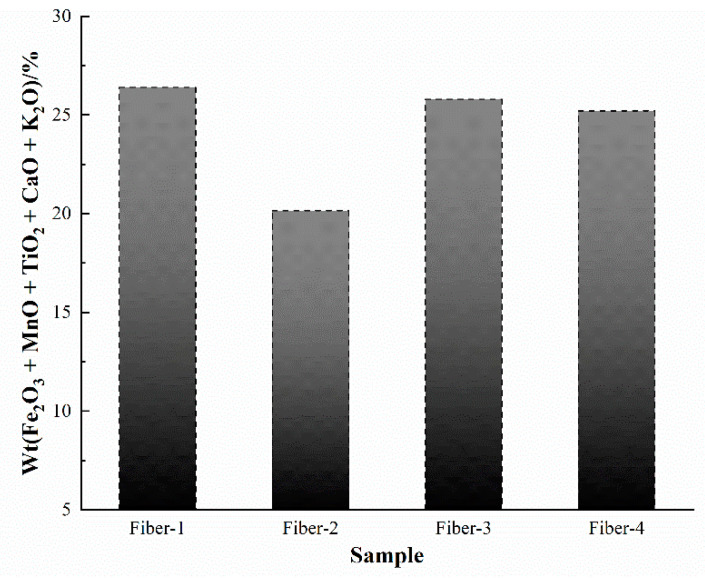
Wt (Fe_2_O_3_ + MnO + TiO_2_ + CaO + K_2_O) of different fibers.

The mass attenuation coefficients of different oxides under gamma ray treatment behave differently. The mass attenuation coefficients of different oxides under the action of electron energy rays in the energy range of 0.01 to 100 MeV was calculated using the XCOM program (Figure 3). The mass attenuation coefficients of different oxides tend to decrease with the increase of radiated quantum energy. When the radiated quantum energy is 0.01~0.2 MeV, the oxides in the fibers can be divided into three groups, based on their corresponding mass attenuation coefficients, namely Fe_2_O_3_ and MnO; TiO_2_, CaO, and K_2_O; and other oxides. The mass attenuation coefficients of the oxides Fe_2_O_3_, MnO, TiO_2_, CaO, and K_2_O, appear significantly higher than those of the other oxides involved. Combined with the oxide component content of different fibers in Table 1, the mass attenuation coefficients of fibers were calculated using the XCOM program (Figure 4). When the radiation quantum energy is 0.01~0.1 MeV, the mass attenuation coefficients of different fibers behave slightly different, among the four samples, Fiber-1 was the lowest, while Fiber-2, Fiber-3, and Fiber-4 were close. This trend is consistent with the content of Wt (Fe_2_O_3_ + MnO + TiO_2_ + CaO + K_2_O) in the fiber (Figure 5). It is observed that the higher the content of oxide components with high mass attenuation coefficients in the fiber, stronger is the radio resistance of the fiber.

In practice, the radio resistance of the fiber ranging between 0.01 to 0.1 MeV can be increased by appropriately increasing oxide content of the fiber with high mass attenuation coefficient in the raw basalt material. However, the impact on the fiber drawing process must be considered while increasing the oxide content. The element content of iron in the basalt fiber generally accounts for about 9–15%. In the process of fiber production, excessive iron content is not conducive to fiber molding, and leads to severe impairment of the platinum rhodium alloy plate used for wire drawing; excessive CaO content increases fiber crystallization temperature and reduces fiber flexibility; the increase in K_2_O and Na_2_O content as alkali metallic elements, reduces the viscosity of the melted basalt and proves detrimental to fiber production. The content of MnO in basalt is only 0.2%, which is often overlooked while examining the properties of basalt fibers, but its mass attenuation coefficient is almost the same as Fe_2_O_3_, which is one of the oxides with the highest mass attenuation coefficient among existing basalt oxides. In addition, the content of TiO_2_ in the fiber itself is also quite low, and increasing its content facilitates the increase in the mechanical property of the fiber, thereby elevating the surface tension and viscosity of the melted basalt, which is conducive to the formation of long fibers [3]. Thus, we conclude that the radio resistance of the fibers can be increased by increasing the content of MnO and TiO_2_ in the raw basalt materials.

### 3.2. Fiber Structures and Surface Characteristics before and after the Irradiation Treatment 

**Figure 6:**
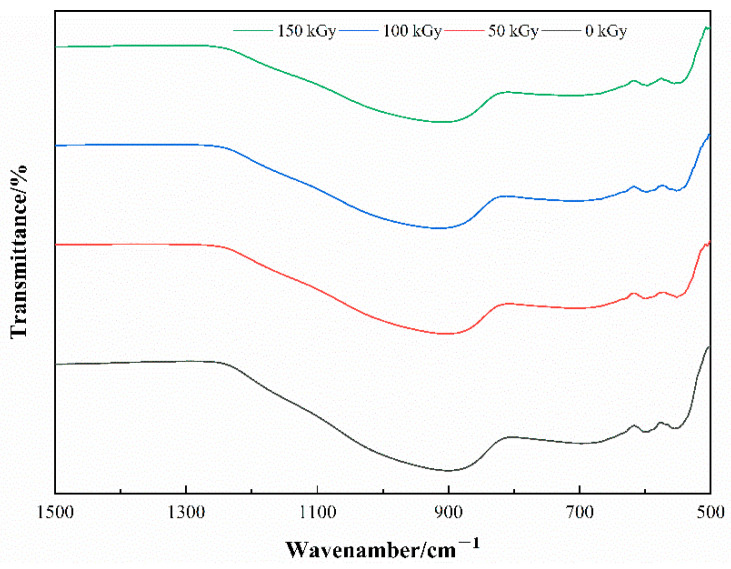
Fiber-1 FTIR characteristics before and after the irradiation.

**Figure 7:**
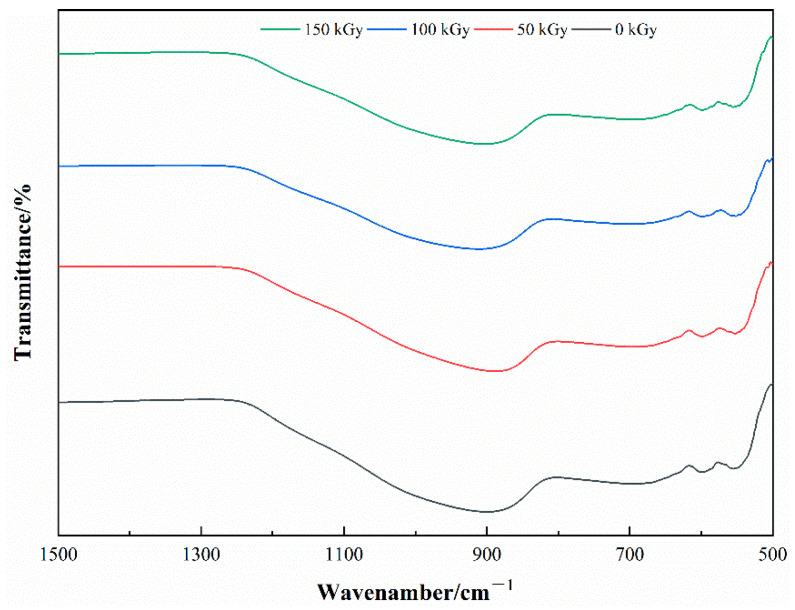
Fiber-2 FTIR Characteristics before and after the irradiation.

To observe the structure, surface morphology, and elemental distribution of characteristics of the basalt fibers before and after the irradiation, the samples, Fiber-1 and Fiber-2, were tested by FTIR, SEM, and EDX. FTIR results showed that there are no absorption bands in region 4000–1500 cm^−1^, therefore, only the region 1500–500 cm^−1^ is shown in Figure 6 and Figure 7.

The FTIR demonstrates that the basalt fiber is a silicate glass with irregular structure. The location and intensity of the absorption bands of the samples with increasing radiation dose exhibit no change and no new absorption bands are created (Figure 6 and Figure 7), and it also illustrates that under the relevant radiation dose used in this experiment, no new chemical bond was formed inside the post-radiation fiber as well as no chemical bond fracture was broken inside the fiber. The strongest absorption band appears near 898 cm^−1^, which is caused by the anti-symmetric stretching vibration of Si-O-Si, and the absorption bond shifts to the lower band, caused by Al element being inserted in the inner structure of the fiber, to form Si-O-Al [26]. The absorption band of 693 cm^−1^ trimmings is a result of symmetric telescopic vibration of the Si-O [20]. The absorption band near 598 cm^−1^ and 551 cm^−1^ is triggered by Si-O bending vibration or stretching vibration of M-O (M is Ca^2+^, Mg^2+^, Na^+^, or K^+^) [27].

**Figure 8:**
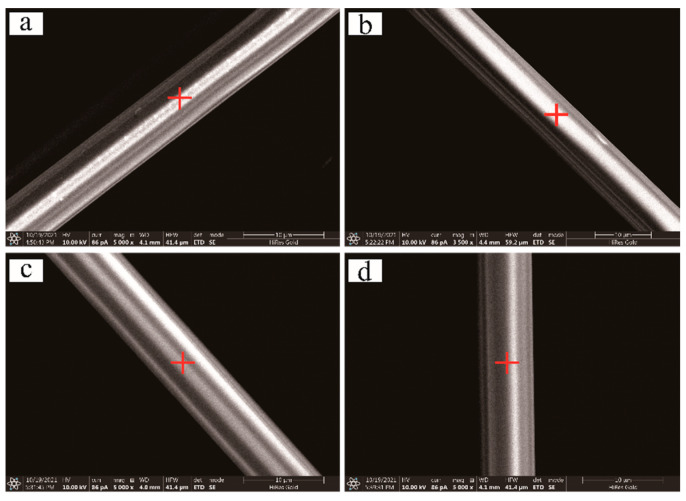
SEM images of the surface of sample fiber-1 before and after the gamma irradiation (before (**a**) gamma irradiation; (**b**) 50 kGy; (**c**) 100 kGy; (**d**) 150 kGy; ➕—EDX test points).

**Figure 9:**
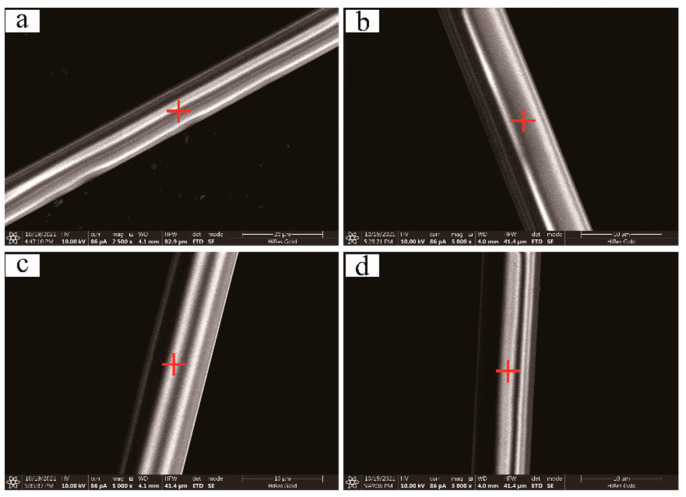
SEM images of the surface of sample fiber-2 before and after the gamma irradiation (before (**a**) gamma irradiation; (**b**) 50 kGy; (**c**) 100 kGy; (**d**) 150 kGy; ➕—EDX test points).

SEM results reveal that the surface of the basalt fiber was smooth and devoid of any defects (Figure 8a and Figure 9a), and even after subjecting to different absorption doses of gamma irradiation, the fiber surface morphology does not change (Figure 8b–d and Figure 9b–d). EDX results establish that the surface elements of the basalt fiber raw filaments are dominated by Si and O, followed by Al, Ca, Mg, Fe, and other elements (Table 2 8a and Table 3 9a), and mostly no change in the content of elements on the fiber surface occurs after gamma irradiation of different doses. Table 2 8b–8d and Table 3 9b–9d, demonstrate that the gamma ray irradiation produces no tangible effect on the distribution of fiber surface elements.

The above experimental results establish that under the radiation absorption dose given in this paper, the structure, surface morphology, and content of the surface elements of the basalt fibers do not demonstrate any considerable changes. This could be attributed to the basalt fiber containing Fe, Ti, Ca, K, Mn, and other elements with high mass attenuation coefficients; specifically, the oxide content of Fe and Ca accounts for about 20% of the total oxide content of the basalt fiber (Table 1), which endows the basalt fiber with good radio resistance. Furthermore, the Si-O structure of Q^4^ dominates within the basalt fiber, and requires higher energy to break down this chemical bond [28].

### 3.3. Changes in Fiber Mass and Mechanical Property before and after the Irradiation

**Figure 10:**
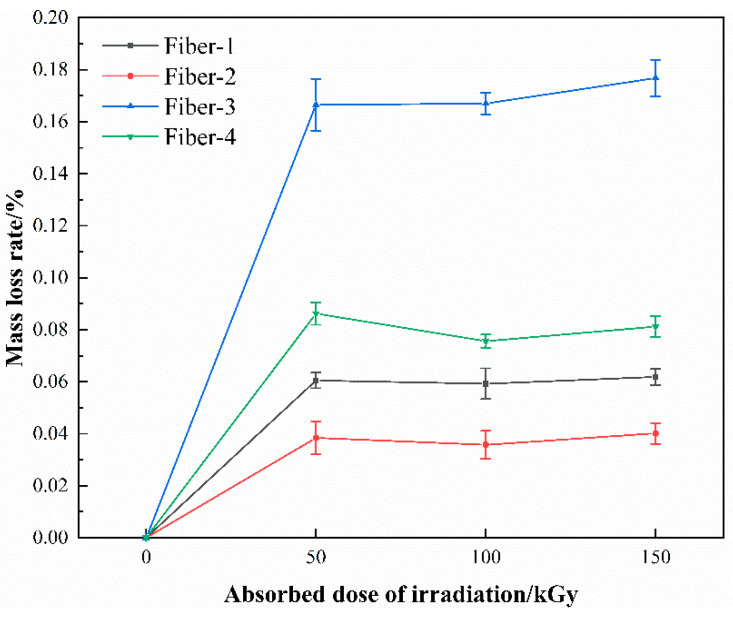
Variation of mass loss rate with the gamma irradiation absorbed dose.

**Figure 11:**
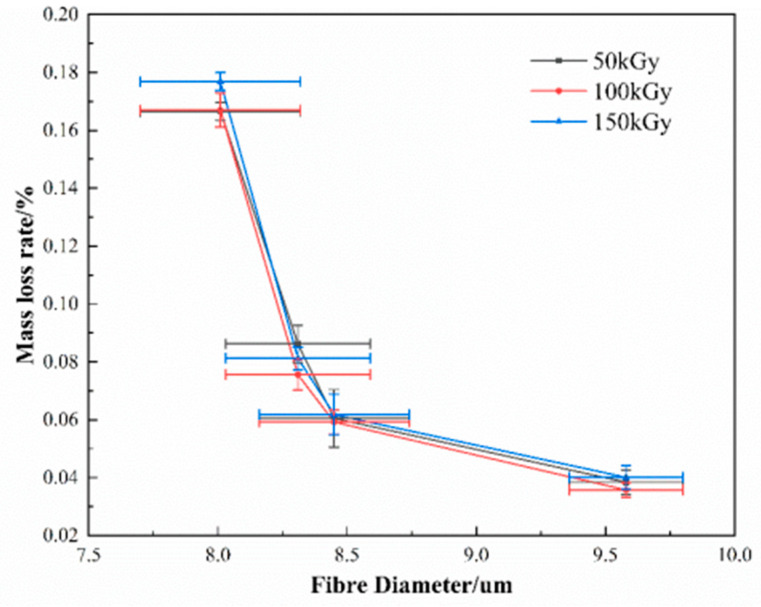
Variation of mass loss rate with fiber diameter.

The mass loss of materials under the radiation environment is an important indicator for estimating its radiation resistance. The test results reveal that the mass loss rates of all the samples increase with the increasing absorption dose, but do not have any significant changes after 50 kGy (Figure 10). The mass loss rates of all the samples for different fibers under the same absorption dose appear different, and the mass loss rates show an increasing trend of Fiber-3, Fiber-4, Fiber-1, and Fiber-2. Illustrated by the XCOM calculation results (Figure 4), Fiber-2 has the lowest fiber mass attenuation coefficient, theoretically, its mass loss rate should be the highest, but the result tends to be the opposite. Fiber-2 has the lowest mass loss rate, indicating that the mass loss of the fiber is not caused by the components losts by the fiber itself. During the analysis it was found that the mass loss rate of a fiber possesses an evident correlation with the diameter of the fiber filament. The smaller the diameter, the greater the mass loss rate of the fiber (Figure 11). Herein, it is deemed that the mass loss of a fiber is caused by the volatilization of gas or water molecules originally adsorbed on to the surface of fiber, which is triggered by the radiation treatment. Research has shown that the smaller the diameter of the fiber, the larger is the specific surface area of the fiber, and the stronger the ability of the fiber adsorbing gas and water molecules, the higher the amount of gas and moisture adsorbed. This also explains the experimental phenomenon that the fiber mass loss rate fails to increase with the increasing radiation dose, even when the radiation dose exceeds 50 kGy. When the absorption dose is 50 kGy, the gas and water molecules adsorbed on the fiber surface evaporate and subsequently, the fiber mass loss rate does not vary further with the increasing absorption dose.

**Figure 12:**
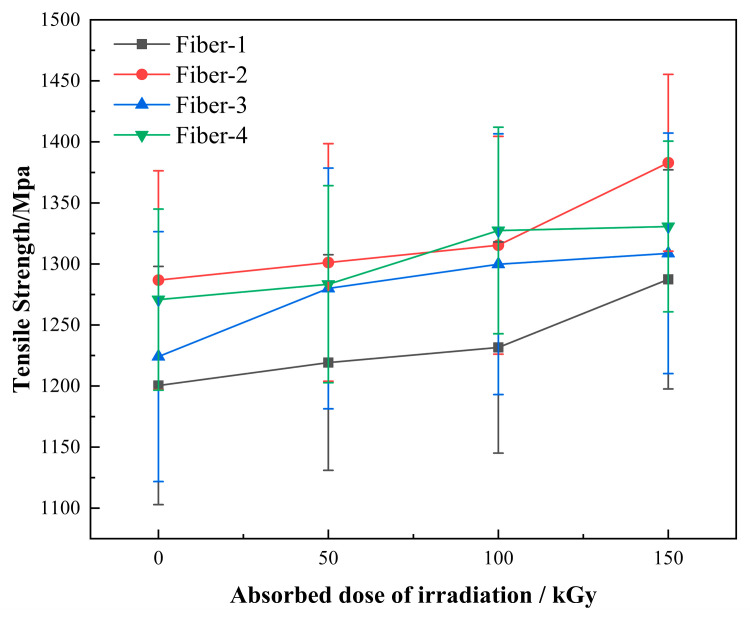
Variation of fiber tensile strength with the gamma irradiation absorbed dose.

**Figure 13:**
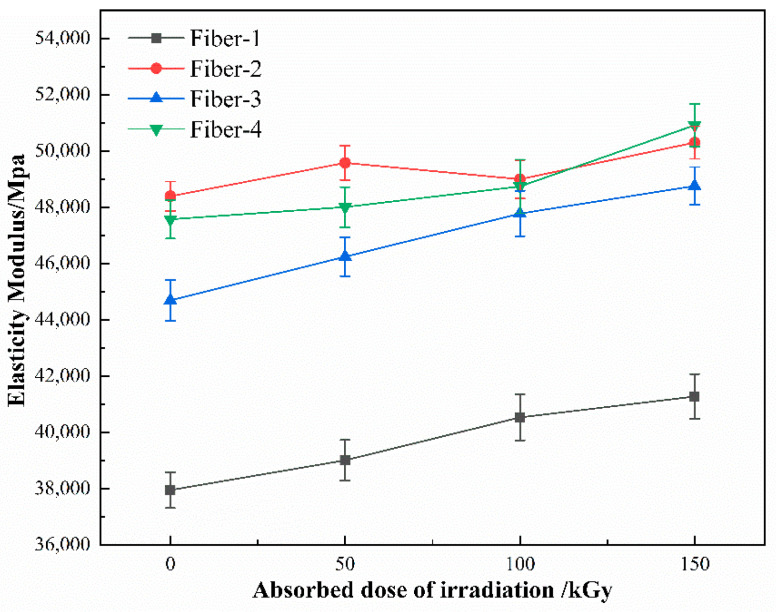
Variation of fiber elastic modulus with the gamma irradiation absorbed dose.

**Figure 14:**
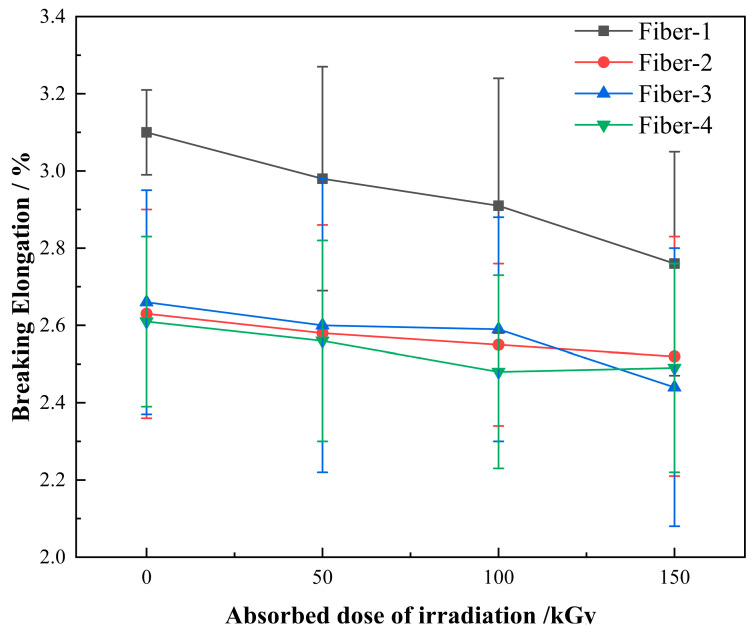
Variation of fiber elongation at break with the gamma irradiation absorbed dose.

Both the tensile strength and elasticity modulus of the fiber increase slightly with the increase of radiation absorption dose (Figure 12 and Figure 13), the increased amplitude of the tensile strength is 4.7–7.5%, and that of the elasticity modulus is 3.9–9.1%. The elongation at break of the fiber decreases slightly with the increase of the absorption dose (Figure 14), and the decreased magnitude ranges within 4.18–10.97%. The experimental results indicate that under a suitable dose of gamma irradiation, the strength and modulus of the basalt fibers can be enhanced to some extent, but the flexibility of the fiber will be reduced. No crystallization occurs within the basalt fiber under the action of gamma irradiation [20], and the experimental results of FTIR, SEM, and EDX reveal that the fiber structure and content of the surface elements do not change either. Hence, considering that water molecules in the fiber are separated from the fiber under the gamma rays, generally, the presence of water molecules will weaken the binding force between other molecules. We speculate that with the separation of these water molecules, the binding force between other molecules in the fiber will increase. The higher the bonding force between the molecules, the higher the strength and modulus of the fiber and the lower the elongation at break.

### 3.4. Fiber Shielding Property

Fiber cloths with different thicknesses possess different shielding effects on the gamma rays of the same energy (Table 4). When the energy was 100 keV, the shielding ratios of the fiber cloths of thickness 0.12 and 0.28 mm were 18.9% and 22.5%, respectively. When the energy was 120 keV, the shielding ratios of the fiber cloths decreased significantly and were at 8.7% and 10.4%, respectively. The conventional radiation shielding material, lead, was selected as a reference, and the shielding ratio of 0.5-mm lead plate experiencing a radiation energy of 100 keV was 48.6% under the same test environment. The fiber cloth thickness was about 1/3 and 1/2 of the lead plate, respectively. However, the shielding ratio of 0.12- and 0.28-mm basalt fiber cloths can reach up to 38.9% and 46.3% of the shielding ratio of 0.5-mm lead plate, respectively, indicating that basalt fiber possesses a good shielding property against gamma rays. As shown in Table 4, the higher thickness of the fiber cloth, the higher the shielding effect; hence, the shielding ratio of basalt fibers can be increased by increasing the thickness of the fiber cloth. Combined with the benefits of basalt fiber in terms of fire and flame retardant properties, mechanical properties, corrosion resistance, etc., basalt fiber cloth exhibits immense potential in the field of gamma ray application.

## 4. Conclusions

The content of each chemical component in the basalt fibers is essentially consistent with the corresponding raw basalt materials. According to the results of oxidation mass attenuation coefficients in the basalt fibers calculated using the XCOM program, the oxides in the basalt fiber can be divided into three groups by mass attenuation coefficients, namely Fe_2_O_3_ and MnO; TiO_2_, CaO, and K_2_O; and other oxides. The different fiber mass attenuation coefficients calculated using XCOM demonstrate that the fiber mass attenuation coefficients are positively correlated with the content of Wt (Fe_2_O_3_ + MnO + TiO_2_ + CaO + K_2_O). Based on the influence of each oxide component in the basalt on the production process, it is believed that the radio resistance of the fibers can be increased by increasing the content of MnO and TiO_2_ in the raw basalt materials. Furthermore, at the radiation dose of 50, 100, and 150 kGy, no change occurs in the surface topography, content of surface elements, and fiber structure of the basalt fibers, indicating that basalt fibers are well resistant to gamma rays. After being subjected to gamma irradiation, the mass loss of the basalt fibers is less than 1%. This mass loss is caused by the volatilization of the gas and water molecules adsorbed on to the fiber surface by the radiation treatment, and not by the loss of the substance of which the fibers are made of. In addition, for the gamma ray irradiation dose used in this experiment, fiber tensile strength and elastic modulus increased 4.7–7.5% and 3.9–9.1%, respectively, but the elongation at break of fiber decreased 4.18–10.97%. When the energy was 100 keV, the shielding ratios of the fiber cloths of thickness 0.12 and 0.28 mm were 18.9% and 22.5%, respectively, but when the energy was 120 keV, the shielding ratios of the fiber cloths decreased significantly and were at 8.7% and 10.4%, respectively. The results indicate that the basalt fiber’s shielding property decreases with the increasing radiated electron energy, and generally, the larger thickness, the better the shielding property of the basalt fiber cloth. When the irradiated electron energy is 100 keV, the shielding ratio for basalt fiber cloths measuring 0.12 and 0.28 mm can reach up to 38.9% and 46.3% of that of the 0.5-mm lead plate, respectively.

On the basis of this study, we believe that basalt fiber has a great application prospect in the field of radiation resistant materials. For example, basalt fibers can be used as reinforcement materials to improve the gamma ray resistance of some composites. Besides, basalt fiber cloth has excellent shield performance to gamma rays, and has the advantage of being light weight, so it has potential application prospect in the field of nuclear protective clothing.

## Figures and Tables

**Table 1:** The results of XRF of basalt and corresponding fiber (wt.%).

SN	Sample Name	SiO_2_	TiO_2_	Al_2_O_3_	Fe_2_O_3_	MnO	MgO	CaO	Na_2_O	K_2_O	P_2_O_5_	LOI	Total
1-1	Rock-1	50.98	3.24	14.21	12.72	0.16	4.49	8.60	2.53	1.62	0.46	1.45	100.47
1-2	Fiber-1	51.11	3.23	14.32	12.51	0.16	4.31	8.79	2.49	1.71	0.47	0.21	99.32
2-1	Rock-2	54.66	1.62	14.52	9.93	0.14	5.90	7.28	3.49	1.03	0.30	0.58	99.45
2-2	Fiber-2	54.79	1.64	14.47	10.11	0.14	6.13	7.12	3.52	1.14	0.29	0.16	99.51
3-1	Rock-3	49.97	4.06	13.16	15.01	0.21	4.15	6.01	4.75	0.34	0.43	2.13	100.21
3-2	Fiber-3	50.19	3.95	13.41	14.97	0.20	4.33	6.32	4.91	0.36	0.44	0.46	99.55
4-1	Rock-4	49.41	1.08	14.82	13.30	0.19	5.82	9.42	2.74	0.57	0.19	1.73	99.29
4-2	Fiber-4	49.97	1.14	15.11	13.47	0.20	5.94	9.81	2.86	0.59	0.20	0.26	99.56

**Table 2:** EDX analysis results of sample fiber-1.

Related Figure Number	Element Content (wt.%)									
	O	Na	Mg	Al	Si	K	Ca	Ti	Mn	Fe
Figure 8a	54.67	0.13	1.26	5.59	23.49	1.23	5.77	1.55	0.08	6.23
Figure 8b	56.31	0.02	0.87	5.38	22.68	0.84	5.76	1.9	0.09	6.15
Figure 8c	53.05	0.01	0.81	6.19	24.32	1.56	5.69	1.43	0.14	6.80
Figure 8d	54.44	0.04	1.02	5.89	24.19	1.13	4.99	1.21	0.12	6.97

**Table 3:** EDX analysis results of sample fiber-2.

Related Figure Number	Element Content (wt.%)									
	O	Na	Mg	Al	Si	K	Ca	Ti	Mn	Fe
Figure 9a	54.15	0.62	1.86	6.86	27.23	0.61	4.03	0.49	0	4.15
Figure 9b	55.82	0.45	1.63	6.19	26.91	0.78	3.93	0.37	0.08	3.84
Figure 9c	54.91	0.59	1.47	5.97	28.01	0.98	3.16	0.53	0.02	4.36
Figure 9d	54.33	0.68	1.72	6.35	26.92	0.69	4.15	0.65	0	4.51

**Table 4:** Shielding ratio of basalt fibers on gamma rays with different energies.

Sample	R/%	
	100 keV/%	120 keV/%
0.12 mm	18.9	8.7
0.28 mm	22.5	10.4
Sheet lead/0.5 mm	48.6	—

## Data Availability

Not applicable.

## References

[1] Fiore V., Scalici T., Di Bella G., Valenza A. (2015). A review on basalt fiber and its composites. Compos. Part B.

[2] Jamshaid H., Mishra R. (2016). A green material from rock: Basalt fiber—A review. J. Text. Inst..

[3] Liu J.Q. (2020). Basalt Fiber Material.

[4] Dhand V., Mittal G., Rhee K.Y., Park S.J., Hui D. (2015). A short review on basalt fiber reinforced polymer composites. Compos. Part B.

[5] Vinotha J.J., Brindha D. (2021). Influence of basalt fibers in the mechanical behavior of concrete—A review. Struct. Concr..

[6] Ni H.C., Arslan M., Qian J.C., Wang Y.P., Liu Z.G., Luo Z.J., Cai R.Q., Gamal E.D.M., Wu Z.R. (2021). Application of basalt fibers in a biological contact oxidation reactor for the treatment of landfill leachate. J. Clean. Prod..

[7] Ralegaonkar R., Gavali H., Aswath P., Abolmaali S. (2018). Application of chopped basalt fibers in reinforced mortar: A review. Constr. Build. Mater..

[8] Tamás D., Tibor C. (2009). Chemical Composition and Mechanical Properties of Basalt and Glass Fibers: A Comparison. Text. Res. J..

[9] Chen X.F., Zhang Y.S., Huo H.B., Wu Z.S. (2020). Study of high tensile strength of natural continuous basalt fibers. J. Nat. Fibers.

[10] Eder H., Schlattl H. (2018). IEC 61331-1: A new setup for testing lead free X-ray protective clothing. Phys. Med..

[11] Wilson J.W., Cucinotta F.A., Miller J.P., Shinn J.L., Thibeault S.A., Singleterry R.C., Simonsen L.C., Kim M.H.Y. (2001). Approach and issues relating to shield material design to protect astronauts from space radiation. Mater. Des..

[12] Little E.A. (2006). Development of radiation resistant materials for advance nuclear power plant. Mater. Sci. Technol..

[13] Li R., Gu Y.Z., Yang Z.J., Li M., Hou Y.W., Zhang Z.G. (2017). Gamma ray shielding property, shielding mechanism and predicting model of continuous basalt fiber reinforced polymer matrix composite containing functional filler. Mater. Des..

[14] Romanenko I., Holiuk M., Nosovsky A., Hulik V. (2018). Investigation of novel composite material based on extra—Heavy concrete and basalt fiber for gamma radiation protection properties. Nucl. Radiat. Saf..

[15] Lazcano M.A.G., Yu W. (2014). Radiative and thermal characterization of basalt fabric as an alternative for firefighter protective clothing. MSAIJ.

[16] Hou Y.W., Li M., Gu Y.Z., Gu Y.Z., Yang Z.J., Li R., Zhang Z.G. (2018). Gamma Ray Shielding Property of Tungsten Powder Modified Continuous Basalt Fiber Reinforced Epoxy Matrix Composites. Polym. Compos..

[17] Zorla E., Ipbu¨ker C., Biland A., Kiisk M., Kovaljov S., Tkaczyk A.H., Gulik V. (2017). Radiation shielding properties of high-performance concrete reinforced with basalt fibers infused with natural and enriched boron. Nucl. Eng. Des..

[18] Hajar S.I., Amin A. (2021). Investigating the effect of basalt fiber additive on the performance of clay barriers for radioactive waste disposals. Bull. Eng. Geol. Environ..

[19] Li R., Gu Y.Z., Yang Z.J., Li M., Wang S.K., Zhang Z.G. (2015). Effect of γ irradiation on the properties of basalt fiber reinforced epoxy resin matrix composite. J. Nucl. Mater..

[20] Yassien K.M., El-Bakary Mohammed A. (2019). Effect of gamma irradiation on the physical and structural properties of basalt fiber. Microsc. Res. Tech..

[21] Wirawan R., Qomariyah N., Ardianto T., Kurniawidi D.W. (2022). Analysis of the radiation parameters of pumice constituent compounds using XCOM. J. Phys. Conf. Ser..

[22] Nikbin I.M., Mohebbi R., Dezhampanah S., Mehdipour S., Mohammadi R., Nejat T. (2019). Gamma ray shielding properties of heavy-weight concrete containing Nano-TiO_2_. Radiat. Phys. Chem..

[23] Yüksel E., Zülfü M.D. (2018). Investigation of usability of limonite aggregate in heavy-weight concrete production. Prog. Nucl. Energy.

[24] Amani A., Al-Buriahi M.S., Rammah Y.S. (2020). Radiation shielding properties of PNCKM bioactive glasses at nuclear medicine energies. Ceram. Int..

[25] Berger M., Hubbell J., Seltzer S., Chang J., Coursey J., Sukumar R., Zucker D., Olsen K. XCOM: Photon Cross Section Database (Version 3.1). http://physics.nist.gov/xcom.

[26] Mostafa N.Y., El-Hemaly S.A., El-Wakeel S.A., Brown P.W. (2001). Characterization and evaluation of the hydraulic activity of water-cooled slag and air-cooled slag. Cem. Concr. Res..

[27] Chen X.F., Zhang Y.S., Hui D., Chen M.R., Wu Z.S. (2017). Study of melting properties of basalt based on their mineral components. Compos. Part B.

[28] Chen Z.W., Huang Y.D. (2016). Mechanical and interfacial properties of bare basalt fiber. J. Adhes. Sci. Technol..

